# Osteoprotegerin-Mediated Homeostasis of Rank^+^ Thymic Epithelial Cells Does Not Limit Foxp3^+^ Regulatory T Cell Development

**DOI:** 10.4049/jimmunol.1501226

**Published:** 2015-08-07

**Authors:** Nicholas I. McCarthy, Jennifer E. Cowan, Kyoko Nakamura, Andrea Bacon, Song Baik, Andrea J. White, Sonia M. Parnell, Eric J. Jenkinson, William E. Jenkinson, Graham Anderson

**Affiliations:** Medical Research Council Centre for Immune Regulation, Institute for Biomedical Research, University of Birmingham, Birmingham B15 2TT, United Kingdom

## Abstract

In the thymus, medullary thymic epithelial cells (mTEC) regulate T cell tolerance via negative selection and Foxp3^+^ regulatory T cell (Treg) development, and alterations in the mTEC compartment can lead to tolerance breakdown and autoimmunity. Both the receptor activator for NF-κB (RANK)/RANK ligand (RANKL)/osteoprotegerin (OPG) axis and expression of the transcriptional regulator Aire are involved in the regulation of thymus medullary microenvironments. However, their impact on the mechanisms controlling mTEC homeostasis is poorly understood, as are the processes that enable the thymus medulla to support the balanced production of mTEC-dependent Foxp3^+^ Treg. In this study, we have investigated the control of mTEC homeostasis and examined how this process impacts the efficacy of Foxp3^+^ Treg development. Using newly generated RANK Venus reporter mice, we identify distinct RANK^+^ subsets that reside within both the mTEC^hi^ and mTEC^lo^ compartments and that represent direct targets of OPG-mediated control. Moreover, by mapping OPG expression to a subset of Aire^+^ mTEC, our data show how *cis*- and *tran*s-acting mechanisms are able to control the thymus medulla by operating on multiple mTEC targets. Finally, we show that whereas the increase in mTEC availability in OPG-deficient (*Tnfrsf11b*^−/−^) mice impacts the intrathymic Foxp3^+^ Treg pool by enhancing peripheral Treg recirculation back to the thymus, it does not alter the number of de novo Rag2pGFP^+^Foxp3^+^ Treg that are generated. Collectively, our study defines patterns of RANK expression within the thymus medulla, and it shows that mTEC homeostasis is not a rate-limiting step in intrathymic Foxp3^+^ Treg production.

## Introduction

An essential feature of thymus function is its capacity to impose self/non-self discrimination, which limits the generation of mature T cells with specificity for self-antigens and the potential to generate unwanted autoimmune responses (1, 2). Both negative selection and the generation of Foxp3^+^ regulatory T cells (Treg) are key to thymic tolerance, and several studies have identified the importance of the thymus medulla, and medullary thymic epithelial cells (mTEC) in particular, in these processes (3–6). The mTEC compartment is known to be heterogeneous and is subject to both positive and negative regulation that ensures the maintenance of organized medullary microenvironments. Positive regulation of the mTEC lineage involves extrinsic signals, which regulate the development and growth of the thymus medulla. Significantly, several members of the TNFR superfamily, including receptor activator for NF-κB (RANK) (7, 8), CD40 (9), and lymphotoxin β receptor (10–13), have been shown to be expressed by mTEC and to play key roles in this process, which involves the provision of RANK ligand (RANKL), CD40L, and lymphotoxin α by hemopoietic cells within the thymus (14–16). In contrast, negative regulation of mTEC numbers involves factors that are intrinsic to mTEC. These include the transcriptional regulator Aire (17, 18) and the soluble mediator osteoprotegerin (OPG) (8, 19), the latter acting as a decoy receptor for RANKL that inhibits RANK-mediated effects.

Many studies have provided insight into the mechanisms regulating the development of the mTEC lineage, including the identification of mTEC progenitors (20–23). Additionally, several reports have revealed complexity within the mTEC compartment with multiple subsets defined by a range of markers, including MHC class II, CD80, Aire, and CCL21. Despite a clearer understanding of the phenotypic makeup of mTEC, the mechanisms that maintain the overall balance of multiple mTEC subsets are not clear. This is of particular significance in relation to the function of the thymus medulla for T cell tolerance induction, where alterations in the mTEC compartment have been shown to be important in the balanced production of conventional and Foxp3^+^ Treg (3, 19). However, although both Aire and OPG are known to influence the mTEC compartment (8, 17, 19, 24), the mechanisms that control mTEC homeostasis and Foxp3^+^ T cell development remain poorly understood.

In this study, we have addressed two issues. First, we have investigated the mechanisms regulating mTEC homeostasis in the adult thymus. Using newly generated reporter mice, we analyzed the mTEC compartment for expression of RANK, a key regulator of medulla formation. We also define the intrathymic source of OPG and map its expression in relation to that of Aire. These experiments identify the RANK^+^ targets of OPG regulation as novel subsets residing within both mTEC^lo^ and mTEC^hi^ compartments. When correlated to detailed analysis of mTEC alterations in mice deficient in either Aire or OPG (*Tnfrsf11b^−/−^*), they explain the broad impact that loss of OPG-mediated control has on the thymus medulla and also identify distinct mechanisms of mTEC control via Aire and OPG. Second, we have studied how alterations in mTEC homeostasis impact Foxp3^+^ Treg development to determine whether mTEC availability is a rate-limiting step in the thymic production of Foxp3^+^ Treg. By generating *Tnfrsf11b*^−/−^ Rag2pGFP mice, we show that the de novo intrathymic generation of Foxp3^+^ Treg is unchanged in the presence of increased mTEC numbers. Such findings show that although Foxp3^+^ T cell development is an mTEC-dependent process, the mechanisms that control the number of Treg produced by the thymus are distinct from the mechanisms controlling mTEC numbers.

## Materials and Methods

### Mice

Adult wild-type (WT) C57BL/6 mice, *Tnfrsf11b*^−/−^ (25), Rag2pGFP transgenic (26), and *Aire*^−/−^ (27) mice were used at 8–12 wk of age. Rag2pGFP mice were crossed with *Tnfrsf11b^−/−^* mice to generate Rag2GFP/*Tnfrsf11b^−/−^* progeny. For the generation of timed pregnancies, the detection of a vaginal plug was set as day 0. All mice were housed at the Biomedical Services Unit at the University of Birmingham in accordance with local and U.K. Home Office regulation. RANK Venus BAC transgenic mice were generated using a genomic BAC clone (BAC RP24-353D23) obtained from the BACPAC Resources Center (Oakland, CA). The fluorescent protein Venus was recombined into the start codon of the *Tnfrsf11a* gene, and DNA was injected into the pronuclei of FVB embryos using standard protocols. Three founder lines were generated that showed evidence of germline transmission. All mouse lines analyzed showed strong levels of Venus expression in thymic epithelial cells, and data shown in the present study are from one representative founder line.

### Cell preparation and thymus digestion

Thymocyte and splenocyte suspensions were produced by mechanical disaggregation. For analysis of thymic stromal cells, adult thymic tissue was enzymatically digested with collagenase dispase (Roche) and DNAse I (Sigma-Aldrich), followed by microbead depletion of CD45^+^ cells (Miltenyi Biotec) as described (28).

### Abs and flow cytometry

For T cell and thymocyte analysis, cells were stained with the following Abs: Brilliant Violet 711 anti-CD4 (RM4-5, BioLegend), Brilliant Violetv510 anti-CD8α (53-6.7, BD Biosciences), allophyocyanin–eFluor 780 anti-TCRβ (H57-597, eBiosceince), and PE/allophyocyanin anti-CD25 (PC61.5, eBioscience). For intracellular staining of Foxp3 alongside GFP preservation, cells were fixed using the BD Cytofix/Cytoperm kit according to the manufacturer’s instructions, and stained with eFluor 450 anti-Foxp3 (FJK-16s, eBioscience). For analysis of thymic B cells, the following Abs (both from eBioscience) were used: anti–CD19-allophycocyanin (MB19-1) and anti-B220 (RA3-6B2, eFluor 450). Isolated thymic stromal cells were stained with the following Abs: allophycocyanin–eFluor 780 anti-CD45 (30-F11, eBioscience), allophyocyanin/PerCP-Cy5.5 anti-EpCAM1 (G8.8, BioLegend), allophyocyanin/PE anti-Ly51 (6C3, eBioscience), BV605 anti-CD80 (16-10A1, BioLegend), and Pacific Blue anti-IA/IE (M5/114.15.2, BioLegend). To analyze CCL21 and Aire expression in TEC from WT, *Aire^−/−^*, and *Tnfrsf11b*^−/−^ mice, intracellular staining of stromal preparations was performed using the Foxp3/transcription factor staining buffer set (eBioscience) according to the manufacturer’s instructions. For analysis of TEC in RANK Venus BAC transgenic mice, the fluorescent protein Venus was preserved using the BD Cytofix/Cytoperm kit. Intracellular staining of cells was performed using the following Abs: Alexa Fluor 488/eFluor 660 anti-Aire (5H12, eBioscience), biotinylated anti-OPG (R&D Systems), and rabbit anti-CCL21 (Lifespan Biosciences), followed by Alexa Fluor 647–conjugated goat anti-rabbit Ab (Life Technologies).

### Fetal thymus organ culture

Freshly isolated embryonic day 15 thymus lobes were placed in organ culture dishes containing 1.35 mM 2-deoxyguanosine, as described (7). After 7 d, fetal thymic organ cultures (FTOC) were stimulated with 10 μg/ml anti-RANK (R&D Systems). After a further 7 d, FTOC were disaggregated with 0.25% trypsin in 0.02% EDTA for TEC analysis by flow cytometry (7).

### Quantitation of Rag2pGFP^+^ recent thymic emigrants

To quantitate thymus output, mechanically prepared spleen suspensions from 8- to 12-wk-old WT Rag2pGFP and *Tnfrsf11b^−/−^* Rag2pGFP mice were subjected to RBC lysis and cells were counted. Cell suspensions were then analyzed by flow cytometry for CD4, CD8, TCRβ, and Foxp3 expression together with Rag2GFP expression, as described above. The number of Rag2pGFP^+^ cells within total CD4^+^ T cells, as well as T conventional cells (CD4^+^Foxp3^−^) and Treg (CD4^+^Foxp3^+^) subsets, was then calculated to determine the frequency of recent thymic emigrants (RTE).

### Immunohistochemistry

Adult thymus tissues were sectioned to a thickness of 7μm, fixed with acetone, and stained for the following Abs: Alexa Fluor 488 anti-Aire (5H12, eBioscience), biotinylated anti-OPG (R&D Systems), and mTEC marker ERTR5 (29) detected with Alexa Fluor 647 goat anti-rat IgM. Images wereacquired using an LSM 780 Zen microscope (Zeiss).

## Results

### Mapping the cellular targets of OPG-mediated mTEC homeostasis in RANK Venus reporter mice

**FIGURE 1.**
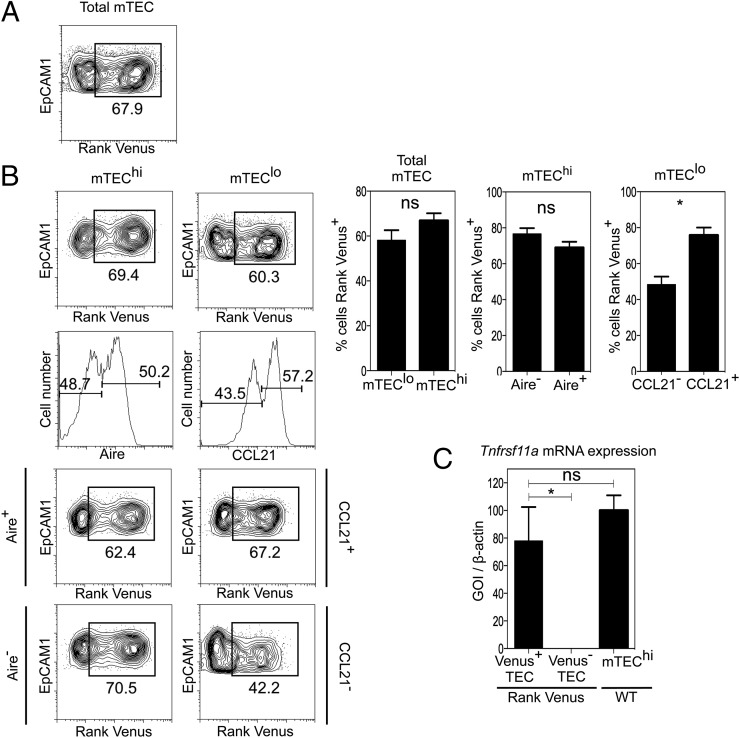
Analysis of RANK Venus reporter mice reveals multiple mTEC targets of OPG-mediated control. CD45^−^EpCAM1^+^Ly51^−^ total mTEC (**A**) from adult RANK Venus reporter mice were analyzed for expression of RANK Venus. Gates are set on RANK Venus negative littermate controls. (**B**) Analysis of RANK Venus expression in total mTEC^hi^ and Aire^+^ and Aire^−^ mTEC^hi^ subsets (*left panels*) and in total mTEC^lo^ cells, and CCL21^+^ and CCL21^−^ mTEC^lo^ subsets (*right panels*). (**C**) RANK Venus expression in TEC isolated from RANK Venus mice correlates with *Tnfrsf11a* mRNA expression, as indicated by quantitative PCR analysis. *Tnfrsf11a* mRNA expression in mTEC^hi^ isolated from WT mice is shown for comparison. Flow cytometry data are typical of three independent experiments using a minimum of three mice per group. All error bars are SEM. *p < 0.05 (unpaired Student *t* test).

The TNFR superfamily member RANK (Tnfrsf11a) plays a key role in the development of Aire^+^ mTEC that regulate tolerance induction via negative selection and Foxp3^+^ Treg generation (8, 9). Importantly, detailed analysis of the mechanisms controlling the thymus medulla has been prevented by an inability to examine RANK expression on a per cell basis within the mTEC compartment. To address this problem, we adopted multiple approaches to examine intrathymic patterns of RANK expression and directly define the cellular targets of OPG-mediated control. First, we generated BAC transgenic mice expressing the fluorescent protein Venus under control of the regulatory elements of the murine *Tnfrsf11a* gene. In these mice, the BAC transgene does not disrupt endogenous *Tnfrsf11a* gene expression, and thymus development and organization are normal (not shown). Analysis of multiple tissues of RANK Venus mice revealed detectable Venus expression in bone, skin, and lymph node, but not in liver, kidney, and lung. Moreover, and as expected by their lack of detectable expression of *Tnfrsf11a* mRNA, flow cytometric analysis showed that thymocytes lacked RANK Venus expression (not shown). In contrast, both Venus^+^ and Venus^−^ cells were readily detectable within the total mTEC population (Fig. 1A), as well as both the mTEC^hi^ and mTEC^lo^ subsets (Fig. 1B). Importantly, when we used FACS to isolate Venus^+^ and Venus^−^ TEC populations from RANK Venus mice, *Tnfrsf11a* mRNA was only detectable in the Venus^+^ cells (Fig. 1C), indicating that Venus expression correlates with *Tnfrsf11a* gene expression.

As further mTEC heterogeneity can be defined by expression of CCL21 (13) and Aire (30), we used intracellular staining methods that did not compromise detection of Venus (data not shown) to analyze expression of these markers within RANK Venus^+^ and RANK Venus^−^ mTEC. Simultaneous analysis of RANK Venus expression with CCL21 and Aire identified additional novel mTEC subsets, including RANK Venus^+^Aire^+^ and RANK Venus^+^Aire^−^ cells within mTEC^hi^, and RANK Venus^+^CCL21^+^ and RANK Venus^+^CCL21^−^ cells within mTEC^lo^ (Fig. 1B). To our knowledge, these findings provide the first direct demonstration of the complexity of RANK expression in the thymus medulla. Moreover, given the role of OPG in the control of RANK-RANKL–mediated development of mTEC, these findings support the idea that OPG controls their homeostasis by operating directly on multiple targets at various stages in the development of the mTEC lineage.

**FIGURE 2.**
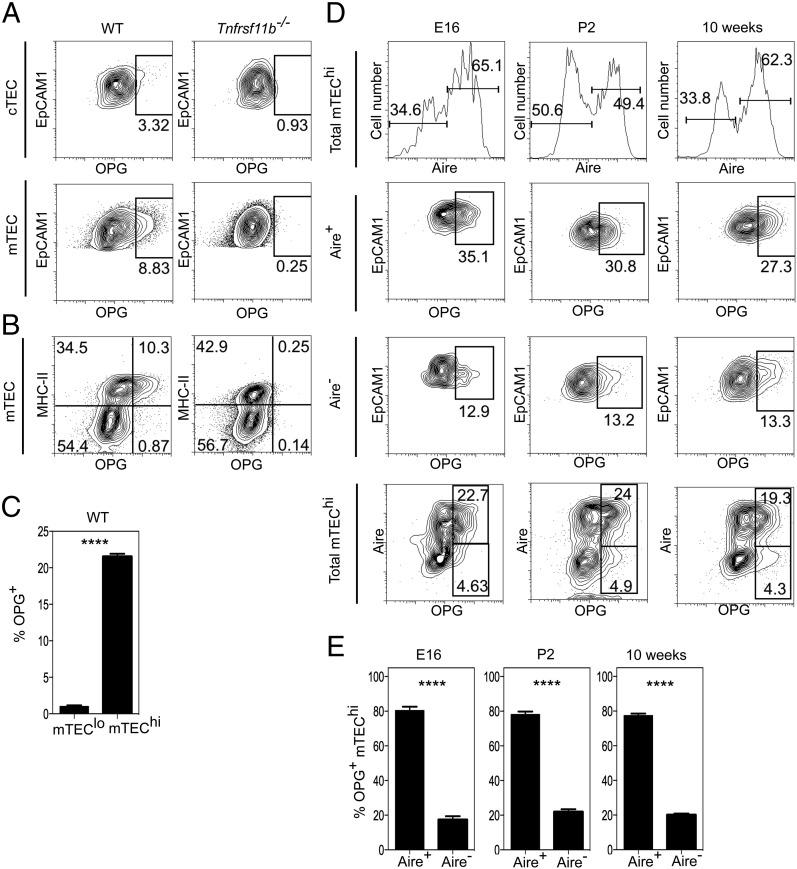
OPG expression defines a subset of Aire^+^ mTEC^hi^ cells. Total cortical TEC (cTEC) and mTEC from adult WT and *Tnfrsf11b^−/−^* mice (**A**) were analyzed by flow cytometry for intracellular expression of OPG. Gates shown are set on levels of anti-OPG staining seen in stromal cells from *Tnfrsf11b^−/−^* mice. (**B**) Flow cytometric analysis of OPG expression in mTEC from WT and *Tnfrsf11b^−/−^* mice, using MHC class II to define mTEC^lo^ and mTEC^hi^ subsets; note the restriction of positive OPG staining to MHC class II^hi^ mTEC^hi^ in WT mice. Statistical analysis of the frequency of OPG^+^ populations in mTEC^lo^ and mTEC^hi^ from WT mice is shown in (**C**). Flow cytometric (**D**) and statistical (**E**) analysis of OPG expression in Aire^+^ and Aire^−^ subsets of mTEC^hi^ at embryonic day 16, postnatal day 2, and in 10-wk-old adult mice. Flow cytometry data shown are representative of at least three separate experiments, and statistical analysis was performed with a minimum of three mice per group. Error bars represent SEM. ****p < 0.0001 (unpaired Student *t* test).

Next, to complement our analysis of RANK expression by mTEC on a per cell basis, we used flow cytometry and confocal microscopy to analyze expression of OPG within the thymus. Freshly prepared stromal populations were stained with Abs to MHC class II and CD80, then permeabilized and stained with Abs to Aire and to OPG. Importantly, to provide appropriate staining levels in control samples, we performed side-by-side analysis of stromal populations from OPG-deficient *Tnfrsf11b^−/−^* mice to allow us to set the staining threshold of the anti-OPG Ab used in this study (Fig. 2A, 2B). In contrast to the background levels of staining seen in stromal cells from *Tnfrsf11b^−/−^* mice, a subset of mTEC in WT mice was OPG^+^ whereas cortical TEC were universally OPG^−^ (Fig. 2A). Further subdivision of adult mTEC into mTEC^hi^ and mTEC^lo^ subsets on the basis of MHC class II (Fig. 2B, 2C) and CD80 expression (not shown) showed that OPG was restricted to a fraction of the mTEC^hi^ population, whereas mTEC^lo^ were uniformly OPG^−^. As the mTEC^hi^ subset is heterogeneous for expression of Aire, and mTEC alterations in *Aire^−/−^* mice indicate its role in the regulation of mTEC development (17, 18, 24), we analyzed expression of both OPG and Aire in the mTEC lineage. Because Aire^+^ mTEC initially emerge in the embryonic period (31), we performed an ontogenetic analysis to determine the pattern of Aire and OPG expression in mTEC during thymus development. Analysis of embryonic and neonatal thymus showed a similar pattern to that seen in the adult thymus, including the predominance of OPG^+^ cells within the first cohort of Aire^+^ mTEC^hi^ in the embryonic thymus (Fig. 2D, 2E). Thus, in both the developing and steady-state thymus medulla, OPG expression defines a subset of Aire^+^ cells in the mTEC^hi^ population.

### OPG and Aire differentially impact mTEC homeostasis

**FIGURE 3.**
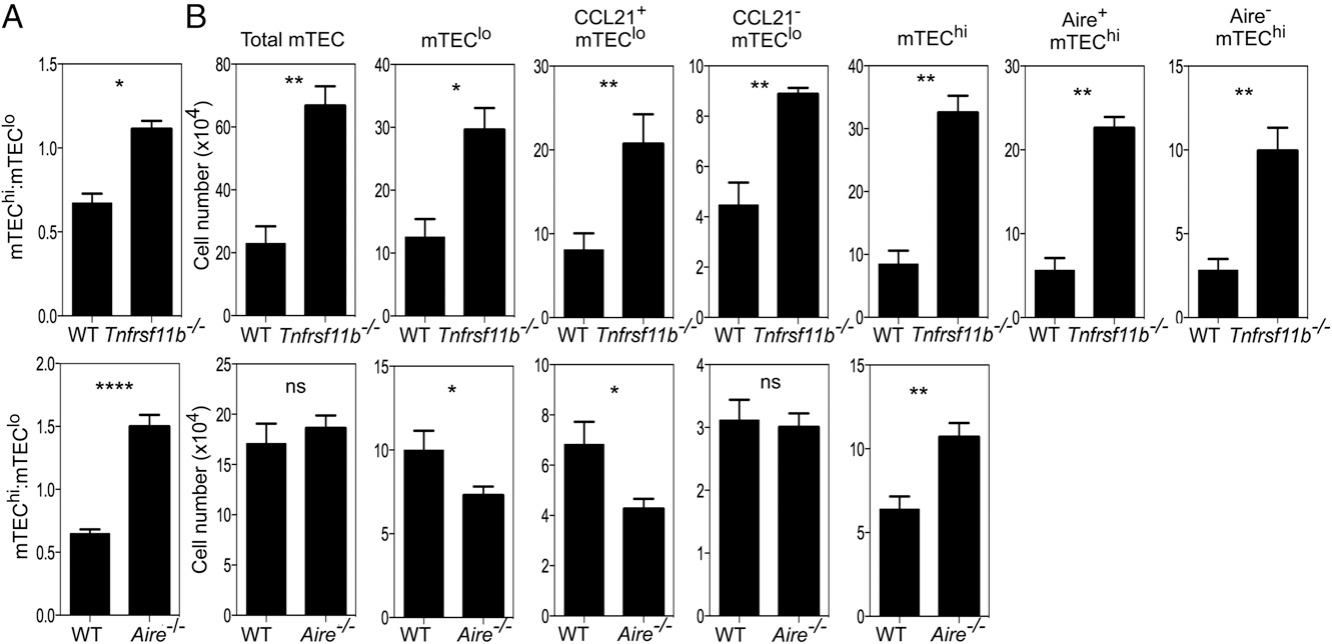
OPG and Aire differentially regulate mTEC homeostasis. Flow cytometric analysis of CD45^−^EpCAM1^+^Ly51^−^ mTEC in adult WT, *Aire^−/−^*, and *Tnfrsf11b^−/−^* mice was performed, and mTEC were subdivided further on the basis of expression of MHC class II and CD80 (mTEC^lo^ and mTEC^hi^), as well as CCL21 and Aire. (**A**) Ratio of mTEC^hi^/mTEC^lo^ in WT and *Tnfrsf11b^−/−^* mice (*upper panel*) and WT and *Aire^−/−^* mice (*lower panel*). (**B**) Quantification of total mTEC and the indicated mTEC subsets in WT and *Tnfrsf11b^−/−^* mice (*top row*) and WT and *Aire^−/−^* mice (*bottom row*). The quantitation of mTEC subsets was performed in three independent experiments, with a minimum of three mice per group. All error bars are SEM. *p < 0.05, **p < 0.01, ****p < 0.0001 (unpaired Student *t* test).

Given the overlap in expression of OPG and Aire, we next performed comparative analysis to assess the impact that the absence of OPG- or Aire-mediated regulation had on the mTEC compartment. To address this, we defined mTEC heterogeneity on the basis of expression of MHC class II, CD80, Aire, and CCL21 in *Aire*^−/−^ and OPG-deficient *Tnfrsf11b*^−/−^ mice. Although both strains demonstrated a skewing in the mTEC^hi^/mTEC^lo^ ratio in favor of mTEC^hi^ (Fig. 3A), *Tnfrsf11b*^−/−^ mice showed a far greater increase in the numbers of both total mTEC and mTEC^hi^ (Fig. 3B). Significantly, *Tnfrsf11b*^−/−^ mice also showed increased numbers of mTEC^lo^, including both the CCL21^+^ and CCL21^−^ subsets, which was not evident in *Aire*^−/−^ mice (Fig. 3B). Thus, despite Aire and OPG being restricted to a subset of mTEC^hi^ (Fig. 2), their absence resulted in a differential impact on the mTEC compartment.

**FIGURE 4.**
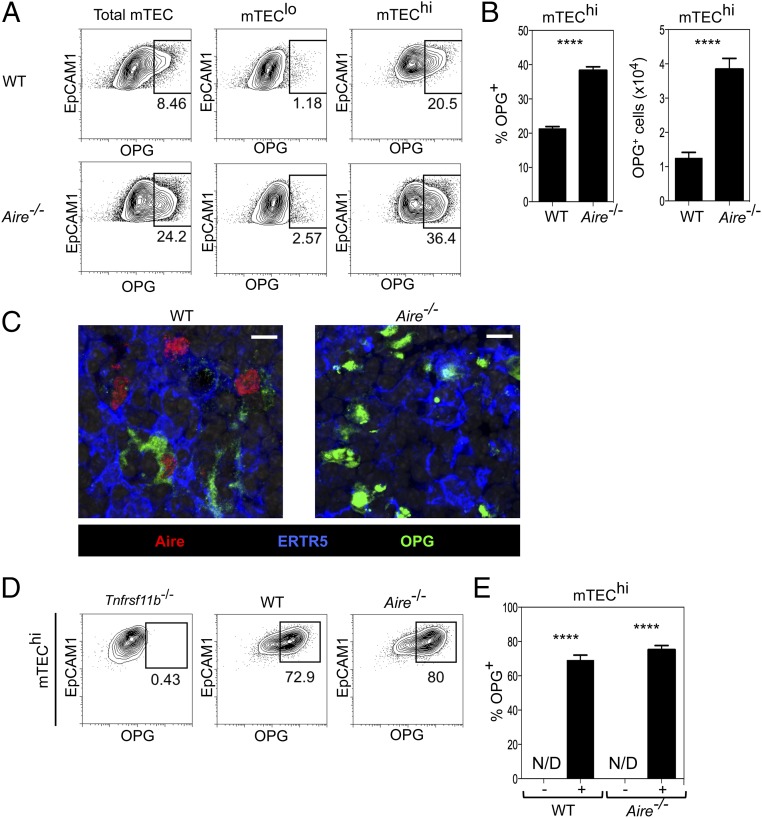
mTEC expression of OPG occurs independently of Aire. (**A**) Flow cytometric analysis of OPG expression in CD45^−^EpCAM1^+^Ly51^−^ mTEC, mTEC^lo^, and mTEC^hi^ in WT (*upper panels*) and *Aire^−/−^* (*lower panels*) mice. (**B**) Quantitation of the number and proportion of OPG^+^ cells in mTEC^hi^ from WT and *Aire*^−/−^ mice. (**C**) Confocal analysis of adult thymic sections from WT (*left*) and *Aire^−/−^* (*right*) mice for expression of Aire, OPG, and the mTEC marker ERTR5. Scale bars, 10 μm. (**D**) Flow cytometric analysis of WT, *Tnfrsf11b^−/−^*, and *Aire^−/−^* dGuo FTOC stimulated with anti-RANK for 7 d. Levels of OPG expression were set on anti-RANK stimulated *Tnfrsf11b*^−/−^ dGuo FTOC. (**E**) Quantitation of proportions of OPG^+^ mTEC^hi^ in untreated (−) or anti–RANK-stimulated (+) WT and *Aire*^−/−^ dGuo FTOC. Data represent at least two independent experiments, with three or more individual mice per group. Error bars represent SEM. N/D, not detected. ****p < 0.0001 (unpaired Student *t* test).

To examine further the relationship between Aire and OPG, we investigated whether the regulation of OPG in mTEC was linked to the expression of Aire. Thymuses from adult WT and *Aire*^−/−^ mice were digested and mTEC^lo^ and mTEC^hi^ were identified by flow cytometry. Fig. 4A and 4B show that OPG^+^ mTEC^hi^ are detectable in both WT and *Aire*^−/−^ mice, demonstrating that Aire is not required for the in vivo expression of OPG by mTEC. This was confirmed by confocal microscopy where OPG^+^ mTEC were detected in both WT and *Aire^−/−^* adult thymus tissue sections (Fig. 4C). Additionally, in vitro anti-RANK stimulation of 2dGuo-treated FTOC from WT and *Aire*^−/−^ embryos showed a similar induction of OPG within mTEC^hi^ in both cases (Fig. 4D, 4E). Interestingly, whereas Aire was not required for expression of OPG, the proportion and number of OPG^+^ mTEC^hi^ actually increased in the absence of Aire (Fig. 4B). Whether this indicates that Aire may act to repress OPG expression is not clear. Alternatively, changes in the lifespan of *Aire*^−/−^ mTEC^hi^ that cause a progressive accumulation of OPG^+^ cells is a further possibility. Whatever the case, our findings identify a subset of Aire^+^ mTEC^hi^ as the cellular source of OPG, a key regulator of mTEC homeostasis, and demonstrate that OPG-mediated control of mTEC homeostasis occurs independently of Aire.

### Foxp3^+^ Treg development is not limited by OPG-mediated mTEC homeostasis

The thymus medulla, and mTEC in particular, are essential in guiding the stepwise generation of Foxp3^+^CD25^+^ thymocytes from their precursors. In contrast, the generation of conventional CD4^+^ thymocytes is mTEC-independent (3). However, the mechanisms that constrain intrathymic Treg development and maintain the balanced production of conventional and regulatory CD4 T cells are unknown. Importantly, direct analysis of the mechanisms controlling this process requires accurate measurement of the intrathymic frequency of newly generated Foxp3^+^ T cells, which is made difficult by the presence of T cells that recirculate back to the thymus from the periphery (32, 33). Indeed, in Rag2pGFP transgenic mice, where GFP^+^ thymocytes represent de novo T cell development, up to half of all Foxp3^+^ cells in the thymus lack GFP expression and include recirculating Treg (33–35).

**FIGURE 5.**
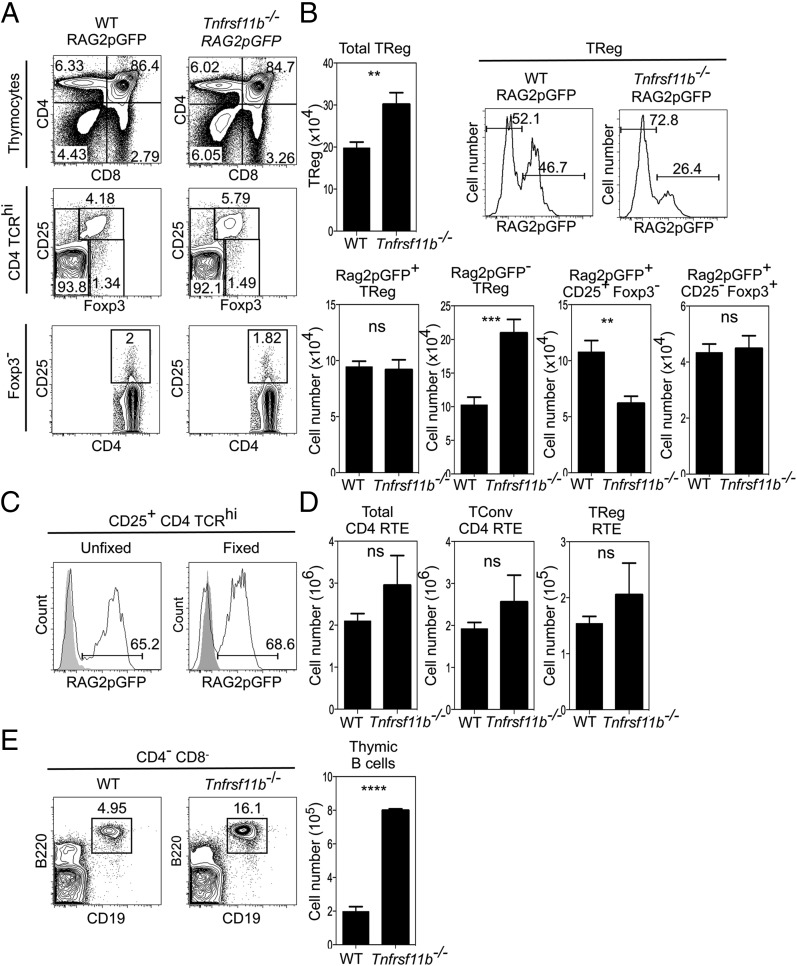
Expansion of the mTEC compartment in *Tnfrsf11b^−/−^* mice does not enhance intrathymic Treg generation. (**A**) Analysis of T cell development, CD25^+^Foxp3^+^ Treg, and CD25^+^Foxp3^−^/CD25^−^Foxp3^+^ Treg precursors in adult WT and *Tnfrsf11b^−/−^* Rag2pGFP mice. (**B**) Quantitation of total thymic Treg, newly generated Rag2pGFP^+^ Treg/precursors, and Rag2pGFP^−^ Treg populations in *Tnfrsf11b^−/−^* Rag2pGFP and Rag2pGFP mice. (**C**) GFP expression is retained after fixation required for intracellular staining of Foxp3. Shaded histograms represent fluorescence levels in WT thymocytes. (**D**) Frequency of total Rag2pGFP^+^ CD4 RTE, Rag2GFP^+^ Treg RTE, and Rag2pGFP^+^ conventional CD4^+^ RTE in the spleens of Rag2pGFP and *Tnfrsf11b^−/−^* Rag2pGFP mice. (**E**) Flow cytometric analysis of the proportion and frequency of thymic B cells in WT and *Tnfrsf11b^−/−^* mice. Data are representative of three separate experiments, and at least three mice per group were used. Error bars show SEM. **p < 0.01, ***p < 0.001, ****p < 0.0001(unpaired Student *t* test).

**FIGURE 6.**
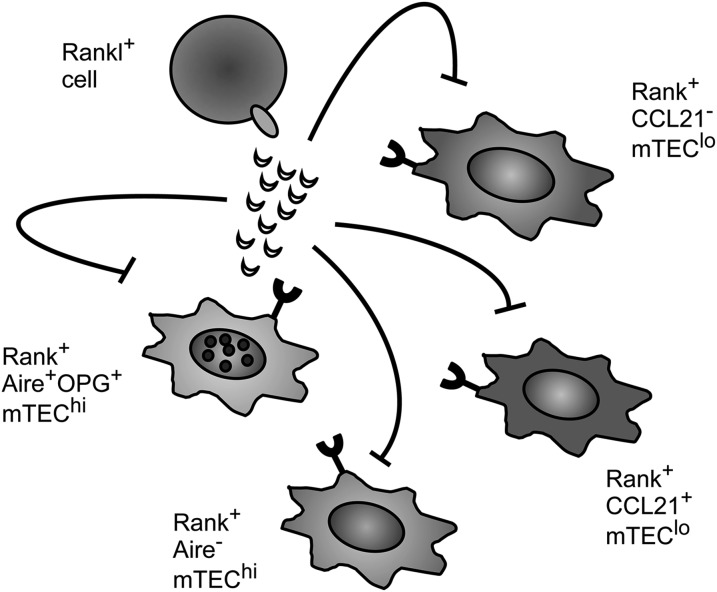
OPG-mediated regulation of RANK signaling during thymus medulla homeostasis involves *cis* and *trans* mechanisms. Development and homeostasis of the mTEC compartment involves interactions between RANKL^+^ hemopoietic cells and RANK^+^ TEC. OPG, the soluble decoy receptor for RANKL, negatively regulates mTEC development by inhibiting RANK-mediated mTEC development. The production and secretion of OPG by a subset of Aire^+^ mTEC, together with the distribution of RANK expression within the mTEC compartment, means that OPG is able to regulate the numbers of multiple RANK^+^ mTEC populations via *cis* and *trans* mechanisms.

To directly examine the impact of increased mTEC availability on the de novo production of Foxp3^+^ T cells from their immature precursors, we crossed Rag2pGFP transgenic mice with OPG-deficient *Tnfrsf11b^−/−^* mice, which show an overall increase in mTEC numbers (Fig. 3). Analysis of conventional CD4^+^ αβT cell development in *Tnfrsf11b^−/−^* Rag2pGFP mice compared with WT Rag2pGFP mice revealed no major alterations in the numbers and proportions of newly generated CD69^+^Qa2^−^ and mature CD69^−^Qa2^+^ single-positive thymocytes (Fig. 5A and data not shown). In contrast, the proportion and number of Foxp3^+^ Treg in *Tnfrsf11b^−/−^* Rag2pGFP mice were increased (Fig. 5A, 5B). To directly quantitate newly generated thymocytes, and so exclude recirculating GFP^−^ T cells, we then analyzed GFP^+^ cells and used anti-Foxp3 staining methods that did not affect detection of GFP (Fig. 5C). Importantly, Fig. 5B shows that the increase in total Foxp3^+^CD4^+^ cells in *Tnfrsf11b^−/−^* mice was not caused by an increase in newly generated Rag2pGFP^+^Foxp3^+^cells, but was caused by an increase in Foxp3^+^ T cells lacking Rag2pGFP expression. Furthermore, analysis of Treg precursor populations (36, 37) showed that the number of Rag2pGFP^+^CD25^−^Foxp3^+^ and Rag2pGFP^+^CD25^+^Foxp3^−^ cells was not increased in *Tnfrsf11b^−/−^* Rag2pGFP mice compared with WT Rag2pGFP controls. In fact, the number of Rag2pGFP^+^CD25^+^Foxp3^−^ cells actually decreased in the absence of OPG (Fig. 5B). Importantly, when we assessed thymic output by measuring the frequency of Rag2pGFP^+^ RTE, no significant differences were observed in the number of Foxp3^+^Rag2pGFP^+^ Treg RTE in the spleens of WT Rag2pGFP and *Tnfrsf11b^−/−^* Rag2pGFP mice (Fig. 5D). Thus, despite increased mTEC availability (Figs. 3, 6), the numbers of Foxp3^+^ Treg that are produced by and emigrate from the thymus are not altered in *Tnfrsf11b*^−/−^ mice, demonstrating that the mechanisms controlling mTEC homeostasis do not determine the efficacy of the intrathymic generation of Foxp3^+^ Treg. Finally, given that the thymus medulla is also known to contain additional cell types, including invariant NKT cells (28, 38) and B cells (39), we analyzed the potential impact that loss of OPG-mediated control of the mTEC compartment had on these populations. Whereas no changes in the makeup of the intrathymic invariant NKT cell pool were detected (not shown), we saw a marked increase in the proportion and absolute number of thymic B cells in the thymus of adult *Tnfrsf11b^−/−^* mice (Fig. 5E). However, although B cells have been recently linked to intrathymic Treg development (40, 41), when taken together with the observation that the generation of Rag2GFP^+^ Treg is unaltered in *Tnfrsf11b^−/−^* mice, this finding suggests that the increased availability of B cells within the thymus does not enhance Foxp3^+^ T cell development.

## Discussion

The thymus imposes T cell tolerance via multiple mechanisms including negative selection and Foxp3^+^ Treg generation (3, 5, 42). The requirement for medullary thymic microenvironments for Foxp3^+^ Treg development is well established, as is the importance of RANK signaling in mTEC development (3, 7, 43). However, whether the mechanisms that control mTEC homeostasis affect the control of intrathymic Foxp3^+^ T cell development is not clear. In our study, we have addressed this issue by analyzing the ways in which two cell-intrinsic regulators of the mTEC compartment, Aire and OPG, control thymus medulla homeostasis.

Our findings show that despite the coexpression of OPG with Aire, their absence affects the mTEC compartment differently. Whereas Aire exerts a relatively modest effect on mTEC^hi^ alone, OPG controls numbers of both mTEC^lo^ and mTEC^hi^. This broad impact that OPG has on the mTEC compartment is consistent with the patterns of mTEC expression of RANK seen in the analysis of RANK Venus reporter mice, which identifies the direct targets of OPG control as novel subsets within both the mTEC^lo^ and mTEC^hi^ populations. Our finding that production of the soluble regulator OPG is limited to a subset of mTEC^hi^ cells, yet RANK expression occurs within both mTEC^lo^ and mTEC^hi^ populations, also indicates that novel *cis*- and *trans*-acting mechanisms operate within the thymic medulla to control mTEC numbers (Fig. 6). These findings provide new insight into how distinct mTEC populations are able to influence one another to control homeostasis within the total mTEC compartment, and they identify further heterogeneity within mTEC that will be important to examine in future work.

Perhaps most significant in relationship to how the thymus medulla controls the balanced production of Foxp3^+^ regulatory and conventional T cells is our finding that negative regulation of mTEC homeostasis does not control the efficacy of intrathymic Foxp3^+^ Treg development. Thus, although mTEC are essential for Treg development, this finding suggests that additional factors that are distinct to microenvironmental regulation and perhaps intrinsic to the T cell lineage also influence the numbers of Foxp3^+^ Treg that the thymus produces. Whereas our data contrast with other studies concluding that increased mTEC numbers enhance Foxp3^+^ thymocyte development (19, 44), it is important to note that these studies did not limit their analysis to de novo Foxp3^+^ Treg generation, as done in the present study. Indeed, we show that the increase in total Treg numbers in the thymus of *Tnfrsf11b^−/−^* mice is caused by an increase in thymus recirculating Foxp3^+^ cells that lack Rag2pGFP expression, and it is not due to changes in newly produced Rag2GFP^+^ Treg. Therefore, the inclusion of mature recirculating Treg that re-enter the thymus from the periphery (32, 33) is likely to explain this discrepancy.

That the loss of OPG-mediated control of the mTEC compartment affects the developmental makeup of the intrathymic Treg pool is of interest given recent reports suggesting that the presence of recirculating Treg in the thymus inhibits Foxp3^+^ T cell development by competing for the availability of IL-2 produced by dendritic cells (45, 46). Significantly, the finding that *Tnfrsf11b^−/−^* mice showed evidence for enhanced circulation of Rag2pGFP^-^ Treg back to the thymus suggests that increased numbers of mTEC may provide more intrathymic niches for recirculating T cells. Interestingly, however, whereas *Tnfrsf11b^−/−^* mice also showed a reduction in Rag2pGFP^+^CD25^+^Foxp3^−^ Treg precursors, this did not lead to a reduction in the number of newly produced Foxp3^+^Rag2pGFP^+^ Treg. Thus, whereas increasing the frequency of mTEC may enhance Treg recirculation that then impacts Treg precursor generation, downstream events such as enhanced compensatory proliferation may take place to maintain de novo Treg production. Additionally, although we find that the frequency of intrathymic B cells is increased in *Tnfrsf11b^−/−^* mice, their lack of detectable RANK Venus expression (not shown) suggests that this is not due to a direct loss of OPG-mediated control of RANK^+^ B cells. Rather, it suggests that mTEC availability regulates the size of the intrathymic B cell population. Although the significance of this is currently unclear, note that whereas B cells have been suggested to play a role in intrathymic Foxp3^+^ T cell development (40, 41), our findings show that increasing their availability does not augment the selection of newly generated Treg. Collectively, our data strongly suggest that mTEC availability does not rate limit intrathymic Treg development. Thus, although studies have shown that Foxp3^+^ T cell development is limited by the number of thymus niches (47, 48), our findings demonstrate that this does not relate to the number of mTEC required for their selection, and rather suggest that the mechanisms controlling Foxp3^+^ Treg generated intrathymically are distinct from those that control mTEC homeostasis.
